# Investigating the neural bases for intra-subject cognitive efficiency changes using functional magnetic resonance imaging

**DOI:** 10.3389/fnhum.2014.00840

**Published:** 2014-10-21

**Authors:** Neena K. Rao, Michael A. Motes, Bart Rypma

**Affiliations:** ^1^Center for Brain Health, School of Behavioral and Brain Sciences, University of Texas at DallasDallas, TX, USA; ^2^Department of Psychiatry, University of Texas Southwestern Medical CenterDallas, TX, USA

**Keywords:** processing speed, intra-subject variability, RT-BOLD, PFC, cognitive efficiency

## Abstract

Several fMRI studies have examined brain regions mediating inter-subject variability in cognitive efficiency, but none have examined regions mediating intra-subject variability in efficiency. Thus, the present study was designed to identify brain regions involved in intra-subject variability in cognitive efficiency via participant-level correlations between trial-level reaction time (RT) and trial-level fMRI BOLD percent signal change on a processing speed task. On each trial, participants indicated whether a digit-symbol probe-pair was present or absent in an array of nine digit-symbol probe-pairs while fMRI data were collected. Deconvolution analyses, using RT time-series models (derived from the proportional scaling of an event-related hemodynamic response function model by trial-level RT), were used to evaluate relationships between trial-level RTs and BOLD percent signal change. Although task-related patterns of activation and deactivation were observed in regions including bilateral occipital, bilateral parietal, portions of the medial wall such as the precuneus, default mode network regions including anterior cingulate, posterior cingulate, bilateral temporal, right cerebellum, and right cuneus, RT-BOLD correlations were observed in a more circumscribed set of regions. Positive RT-BOLD correlations, where fast RTs were associated with lower BOLD percent signal change, were observed in regions including bilateral occipital, bilateral parietal, and the precuneus. RT-BOLD correlations were not observed in the default mode network indicating a smaller set of regions associated with intra-subject variability in cognitive efficiency. The results are discussed in terms of a distributed area of regions that mediate variability in the cognitive efficiency that might underlie processing speed differences between individuals.

## INTRODUCTION

Experimental psychology research has identified basic speed-based processing resources that govern an individual’s consistent performance (i.e., speed and accuracy) across a broad range of cognitive tasks compared to other individuals. This *inter*-subject variability in processing speed resources contributes to individual differences in general intelligence, *g* (Spearman, 1904; Kahneman, 1973; Vernon, 1983; Just and Carpenter, 1992). Processing-speed theories posit that individual differences in *g* arise from variability in the efficiency with which fundamental cognitive operations are performed (Vernon, 1983). These theories posit a central role for cognitive efficiency on the idea that when cognitive operations can be performed quickly, neural resource allocation can be minimized, made available for other task-relevant cognitive operations, and performance can be maximized (i.e., “the limited time principle,” Vernon, 1983; Salthouse, 1996). Notably, just as some individuals are more consistent in their performance compared to other individuals across a broad range of cognitive tasks, some individuals also are more consistent in their performance from one trial to another. This *intra*-subject performance variability also is associated with variability in *g* (Vernon, 1983) and can reflect alterations at a systems or cellular level in the brain and possibly indicate underlying pathology (MacDonald et al., 2006).

Intra-subject variability in processing speed indexes unique aspects of cognition compared to inter-subject variability (Jensen, 1992a). For example, although reaction time (RT) SD generally increases with mean RT on a variety of cognitive tasks, RT SD predicts unique variance in Raven’s Progressive Matrix scores (i.e., a measure of *g*) relative to mean RT (Jensen, 1992a). Additionally, greater RT variability is associated with lower Intelligence Quotient [IQ as measured by Wechsler Adult Intelligence Scale (WAIS-III-R); Wechsler, 1985; Jensen, 1992b]. Physiological research suggests a plausible neural basis for individual differences in processing speed task performance. Faster central nervous system (CNS) nerve conduction velocity (NCV) predicts higher *g* (Reed and Jensen, 1992), and greater intra-subject variability in CNS NCV contributes to greater RT variability and lower IQ (Barrett et al., 1990) even though inter-subject variability in peripheral nervous system NCV is not a reliable predictor of *g* (Barrett et al., 1990; Barrett and Eysenck, 1993; Rijsdijk et al., 1995; McRorie and Cooper, 2004). Additionally, Burzynska et al. (2013) observed greater structural integrity, as measured by white matter tracts, was associated with a more efficient use of task-related gray matter processing resources in task-positive regions on an n-back working memory task.

Behavioral evidence for individual differences in cognitive efficiency relies on measures of processing speed (e.g., Vernon, 1983; Salthouse, 1996) such as the Digit-Symbol Coding task from the WAIS-III-R (Wechsler, 1985). They are designed to assess the time required to perform elementary cognitive operations. While such tasks are designed to be sufficiently simple to minimize the influence of semantic knowledge, memory, and strategy on performance, the imposition of time-limits in these tasks allows them to be sufficiently complex so as to measure more than just sensorimotor functions (see Carroll and Maxwell, 1979; Vernon, 1983; Jensen, 1993; Salthouse, 1996). Measurement of the execution speed of one or a few isolated cognitive processes (e.g., visual search, response selection) is thought to index the efficiency with which more complex operations (e.g., reading comprehension, motor-sequence learning) are performed. This execution speed has been posited to reflect the integrity of physiologic mechanisms (i.e., the “neural efficiency”; Vernon, 1983; Jensen, 2006) such as the extent of myelin content (see Glasser and Van Essen, 2011) or its function (e.g., Buxton et al., 2004; Attwell et al., 2010; Hutchison et al., 2013).

Neuroimaging results suggest that the frontal cortex mediates inter-subject differences in cognitive efficiency (Rypma et al., 2006; Motes et al., 2008, 2011; Zhu et al., 2013). For instance, when participants perform a fMRI-adapted version of the Digit-Symbol Coding task (Wechsler, 1985), faster participants show less task-related BOLD percent signal change in dorsal prefrontal cortex [PFC; Brodmann’s Areas (BAs) 9 and 46] than slower participants, but faster participants show more task-related BOLD percent signal change in ventral PFC (BA 44) and parietal regions (BAs 39 and BA 40). Additionally, in young adults, faster performers show less dorsal PFC functional connectivity with other regions during the task than slower performers (Rypma et al., 2006; Rypma and Prabhakaran, 2009; Kannurpatti et al., 2011) suggesting that PFC plays a central role in speed-related functions by exerting greater executive control in the presence of slower and less accurate performance (see Rypma and Prabhakaran, 2009). Additionally, young adults show lower task-related BOLD percent signal change associated with faster speeds on processing speed within the PFC; whereas older adults show higher task-related BOLD percent signal change associated with faster speeds within PFC (e.g., Rypma and D’Esposito, 2000; Motes et al., 2011) indicating PFC-mediated age-related differences in processing speed. Additionally, age-related increases in frontal brain activity are associated with poorer task-switching performance among the elderly (Zhu et al., 2013).

Given that intra-subject variability indexes unique cognitive components compared to inter-subject variability (Jensen, 1992a) and in fact is a common component of cognitive decline or behavioral changes associated with aging, traumatic brain injury, attention-deficit hyperactivity disorder, and schizophrenia (MacDonald et al., 2006), we set out to explore the neural regions contributing to this variability in young adults. In the present study, we explored the extent to which intra-subject differences in processing speed were related to neural activity in healthy young adults. We used an fMRI-adapted Digit-Symbol Verification Task (DSVT; see Rypma et al., 2006) to examine trial-by-trial RT correlations with BOLD percent signal change. It is known that faster participants show less BOLD activity than slower participants in task-positive regions (Haier et al., 1988; Rypma and D’Esposito, 1999; Rypma et al., 2006; Motes et al., 2011). Thus, we expected to observe positive trial-level RT-BOLD correlations in which faster RTs would be associated with less BOLD percent signal change in a subset of regions showing DSVT task-related involvement. Observation of such performance-level correlations will lend insight to region-specific mechanisms of neural cognitive efficiency.

## MATERIALS AND METHODS

### PARTICIPANTS

Thirty participants (ages 20–39 years, 13 M, 27 right-handed) were recruited from the University of Texas at Dallas campus through advertisements. Participants were excluded if they had any MRI contra-indicators, a history of brain trauma, neurological or psychiatric disorders, or if they were taking psychotropic drugs. The experiment was approved by the Institutional Review Boards for University of Texas at Dallas and University of Texas Southwestern Medical Center, and the experiment was conducted according to the principles expressed in the Declaration of Helsinki. All participants gave informed, written consent prior to participating.

### PROCEDURE

**FIGURE 1:**
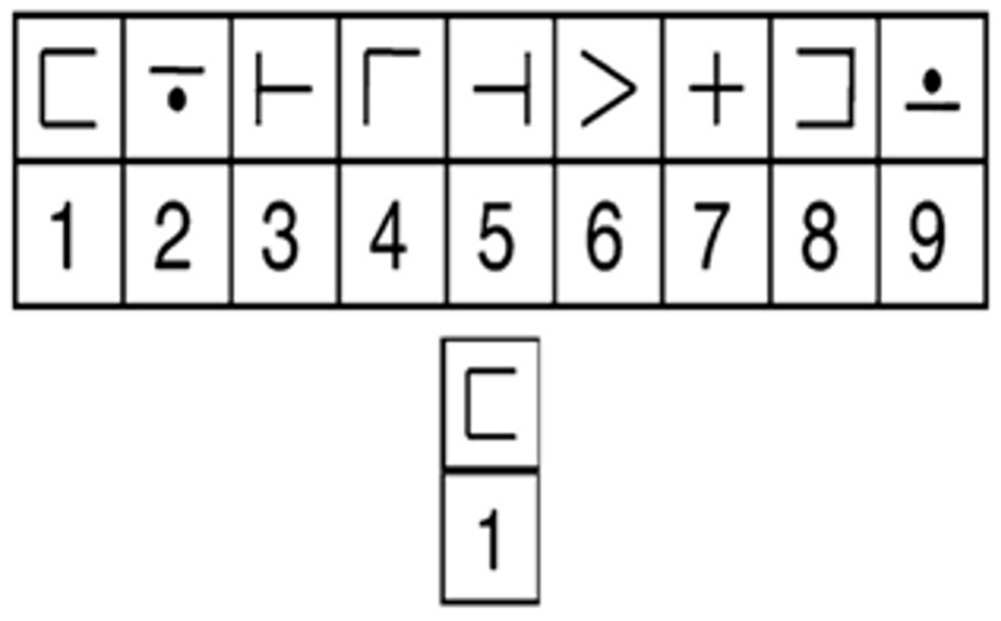
**Example of a DSVT trial.** Stimuli consisted of an array of digit-symbol pairs (nine simple shapes each paired with a single digit, one through nine) and a single digit-symbol probe. The array and probe were centered on the screen and presented for 3.5 s. While undergoing fMRI scanning, participants indicated whether the probe was present (right button-press) in the array or absent (left button-press). Rest periods (0.5, 4.5, 8.5, or 12.5 s; *n* = 23 per run) followed each trial. Novel digit-symbol pairings appeared on each trial.

While undergoing fMRI scanning, participants completed a modified version of the digit-symbol verification task (DSVT; see Rypma et al., 2006), which was adapted from the Digit-Symbol Coding test from the WAIS-III-R (Wechsler, 1985). An array of digit-symbol pairs (nine simple shapes each paired with a single digit, one through nine) and a single digit-symbol probe-pair (**Figure 1**) appeared simultaneously for 3.5 s. Participants were instructed to indicate with a button-press on MR compatible button-boxes if the probe was present (right thumb button-press) in the array or absent (left thumb button-press) from the array. The probe was present in half of the trials and absent in the other half. The order of the trials was randomized throughout each run, as was the numeric location of the digit-probe in the array. Participants were given until the end of the display time to respond, but were instructed to respond both as quickly and as accurately as possible. RT was recorded from the onset of the stimulus until the participant responded. Rest periods (0.5, 4.5, 8.5, or 12.5 s; *n* = 23 per run) were randomly intermixed between trials to give a jittered event-related trial design. Participants completed three runs with 52 trials per run (a total of 156 trials over the three runs). Digit-symbol pairings in the array varied across the trials to avoid learning or memory-based strategies.

Stimuli were projected onto a screen at the rear of the bore of the scanner and were viewed by the participants via an angled mirror (∼45°) positioned above the receiving coil, with the midpoint of the mirror approximately 12 cm from a participant’s eye. E-prime (Psychology Software Tools, Pittsburgh, PA, USA) was used to control stimulus presentations and to record RT and accuracy.

Following MRI acquisition and outside of the scanner, participants completed the Digit-Symbol Coding Task from the WAIS-III-R (Weschler, 1985).

### MRI ACQUISITION

Image acquisition was performed using a 3T MRI scanner (Siemens) equipped with a standard head coil (8-element, SENSE, receive-only). Foam padding was used to prevent head motion. High resolution MPRAGE anatomical images (TR = 3.7 ms, resolution = 1 mm isovoxel, flip angle = 12°, slices = 160 saggital orientation) and three functional EPI runs (TR = 2000 ms, TE = 30 ms, resolution = 3.5 mm × 3.5 mm × 4 mm, flip angle = 70°, slices per volume = 45, volumes per run = 150) were acquired for each participant. Before each functional run, nine whole-volume EPI scans were run to remove any T1 saturation effects and discarded prior to analysis.

### DATA ANALYSIS

Data were analyzed using AFNI software (Cox, 1996). Images for each participant were first corrected for slice-timing and head motion offsets. Participants included in these analyses had movement of <1 mm. These data were corrected by registering each 3D sub-brick from each run to the base sub-brick of the first functional run (Cox, 1996). Additionally, we included motion parameters (i.e., roll, pitch, yaw, x, y, z) in the deconvolution analysis to remove these effects from the subject-level data. Data were then spatially smoothed with a Gaussian kernel (FWHM = 8mm). At each voxel, data then were expressed in terms of percent signal change relative to the mean (i.e., 100^∗^ yt/My, *t* = time point) per run so that the deconvolution parameter estimates would be expressed in terms of percent signal change.

We used a modulated deconvolution neuroimaging technique (Cox, 1996) to investigate the neural basis of intra-subject differences in processing speed performance. FMRI studies have shown the efficacy of including behavioral measures of trial-to-trial variability in cognitive processes while modeling BOLD responses to observe intra-subject differences (see MacDonald et al., 2006). An organized difference in RT can contribute to variability in activation patterns and represent a longer engagement of some processes (Christoff et al., 2001). Thus, modeling such auxiliary behavioral information in the analyses can provide more information. Hemodynamic response function (HRF) regressors modulated by trial-level RTs have the potential to better account for BOLD percent signal changes than the more typical non-modulated models, thus increasing statistical power (Grinband et al., 2008). RT-modulation analyses have been used to explore a broad range of cognitive processes including selective attention (Weissman et al., 2006), spatial attention (Prado and Weissman, 2011), inhibition (Bellgrove et al., 2004), as well as shared processes across a range of cognitive tasks (Yarkoni et al., 2009).

For each participant, voxel-wise analyses were carried out to obtain (1) performance-independent DSVT-related BOLD percent signal change and (2) performance-dependent trial-level RT-BOLD correlations using amplitude modulated linear deconvolution analyses (Cox, 1996).

#### Performance-independent DSVT effects

**FIGURE 2:**
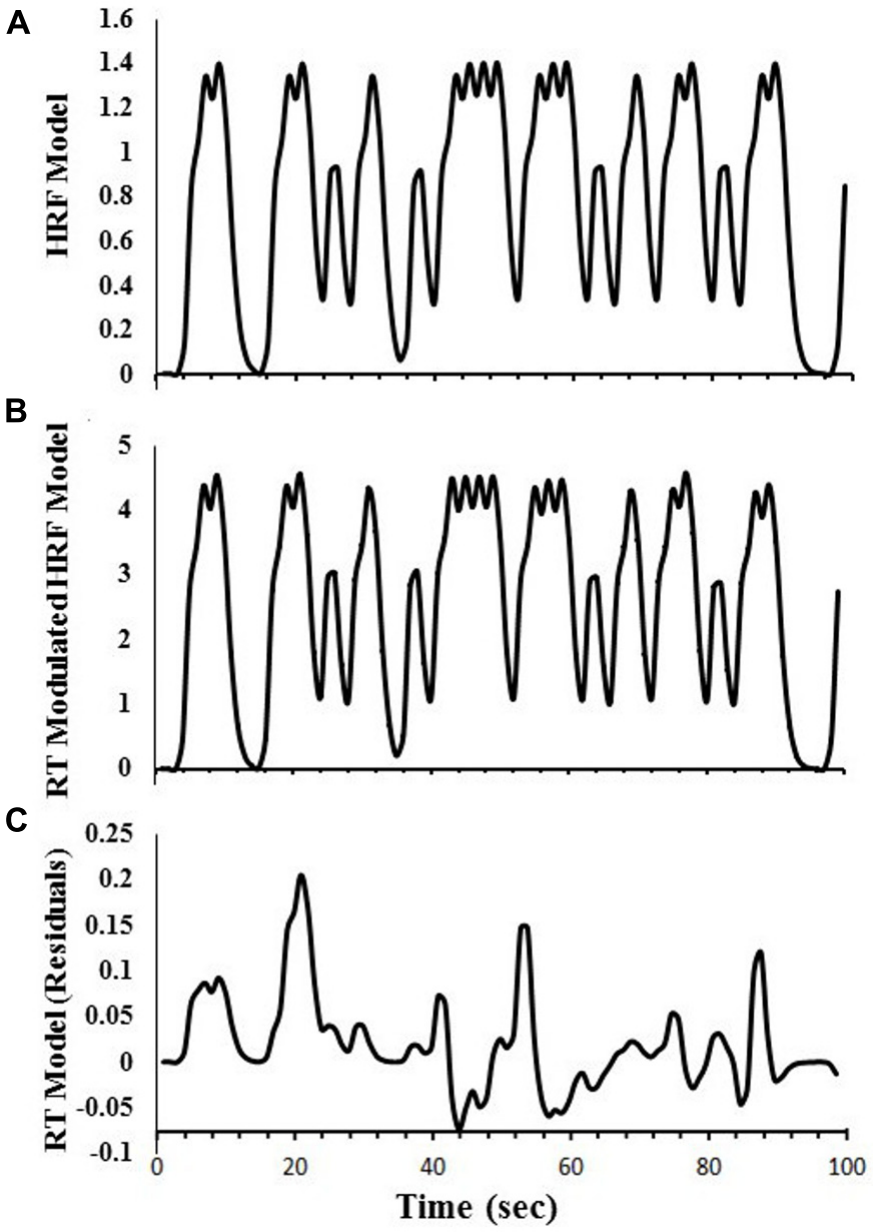
**Examples of regressors created for the amplitude modulated linear deconvolution of the time-series.** Data are based on a single run for one study participant. **(A)** The canonical HRF model was first created to obtain estimates of DSVT-related percent signal-changes. A gamma-variate function was convolved with trial onset times (for correct responses only). **(B)** Each gamma-variate function in the model HRF was scaled based on corresponding standardized trial-RTs [i.e., log(RT)]. **(C)** The RT-Modulated HRF model was regressed on the HRF model to remove the canonical HRF effects and obtain the RT model time-series. See “Materials and Methods” section for equations.

Regressors were created to obtain estimates of DSVT-related BOLD percent signal changes for correct responses only. For the DSVT effects, a canonical HRF [i.e, a gamma-variate function (Cohen, 1997) with parameters *b* = 8.6, *c* = 0.547; max amplitude = 1.0] was convolved with trial onset time-courses for correct responses only (e.g., **Figure 2A**). This regressor was regressed on subjects’ data to identify brain regions where BOLD percent signal change varied with the task (DSVT effect Bs).

$$y_{D\,S\,V\,T}\,\left(t\right)=\sum_{k=1}^{K}h\,\left(t-\tau_{k}\right),\;w\,h\,e\,r\,e\,\;h_{t}=t^{8.6}\,\exp\left(-t/0.547\right)$$

#### Performance-dependent trial-level RT effects

Another regressor also was created to obtain RT variability effects (i.e., trial-level processing speed effects for correct responses only). That is, the DSVT-effect regressors based on the canonical HRFs (i.e., **Figure 2A**) were proportionally scaled based on the corresponding standardized trial-RTs (e.g., **Figure 2B**). Standardized trial-RTs were calculated by taking the logarithm of each RT (Ratcliff, 1993).

$$y_{D\,S\,V\,T-R\,T\,M\,O\,D}\,\left(t\right)=\sum_{k=1}^{K}h\,\left(t-\tau_{k}\right)*\left[\log\left(R\,T\right)\right]$$

These RT-scaled HRF models (i.e., **Figure 2B**) were regressed on the canonical HRF-based regressors (i.e., **Figure 2A**) to remove the canonical HRF effects and produce a residual standardized trial-level RT time-series (e.g., **Figure 2C**). This trial-level RT time-series regressor was regressed on subjects’ data to identify regions where trial-level RTs were correlated with trial-level BOLD percent signal change (trial-level DSVT-RT effect Bs).

$$y_{R\,T}\,\left(t\right)=y_{D\,S\,V\,T-R\,T\,\;M\,O\,D}\,\left(t\right)-y_{D\,S\,V\,T}\,\left(t\right)$$

Each participant’s 3D structural image (co-registered to the functional data) was transformed, via a 12-parameter affine transformation, to fit it to a Talairach template (i.e., the Colin-brain template; Talairach and Tornoux, 1988). The B-maps for all conditions were transformed to Talairach space based on structural transformation parameters. Group whole-brain one-sample *t*-test analyses were carried out to identify brain regions where BOLD percent signal change (DSVT effect Bs) varied with the task and where trial-level BOLD percent signal change varied with trial-level RTs (intra-subject DSVT-RT effect Bs) to observe intra-subject variability in processing speed.

All results were cluster thresholded based on Monte Carlo simulations (AlphaSim; Ward, 2000) so that Family Wise Type 1 α ≤ 0.05 (145 voxels at 2 mm isovoxel resolution with defining neighboring voxels as being connected by surfaces, edges, or corners).

## RESULTS

### BEHAVIORAL RESULTS

Overall, participants made few errors (*M* = 94.2% accurate, SD = 2.3%), and their RTs (M = 1696 ms, SD = 273ms; see **Table 1** for behavioral data) were comparable to previous findings (Rypma et al., 2006). Participants’ mean RT (*M* = 1696 ms, SD = 273 ms) was significantly correlated with participants’ SD RT (*M* = 379.60 ms, SD = 96.38 ms; *r* = 0.84, *p* < 0.001) suggesting larger variability in RT was associated with poorer, or slower, mean RT (i.e., performance). RT and accuracy were significantly negatively correlated, (*r* = -0.61, *p* < 0.05) indicating that faster participants were also more accurate. Thus, suggesting participants were not randomly responding and that speed-accuracy trade-off was not an issue at the group level.

**Table 1:** Digit-symbol coding and DSVT behavioral data (mean and SD) and correlations to digit-symbol coding.

	Mean	SD	Digit-symbol coding number correct correlation [*p* value (2-tailed)]
Digit-symbol coding number correct	90.30	10.36	–
DSVT accuracy total	94.22%	2.25%	0.45 (*p* = 0.01)
DSVT mean RT (ms) total	1695.92	272.94	-0.52 (*p* = 0.003)
DSVT SD RT (ms) total	379.60	96.38	-0.64 (*p* < 0.001)

Participants’ performance on the Digit-Symbol Coding task (M_numbercorrect_ = 90.3, SD = 10.4) was significantly correlated with all measures from the DSVT (**Table 1**). Thus, this provides convergent validation for this fMRI-adapted DSVT as a measure of processing speed and cognitive efficiency.

### fMRI RESULTS

**FIGURE 3:**
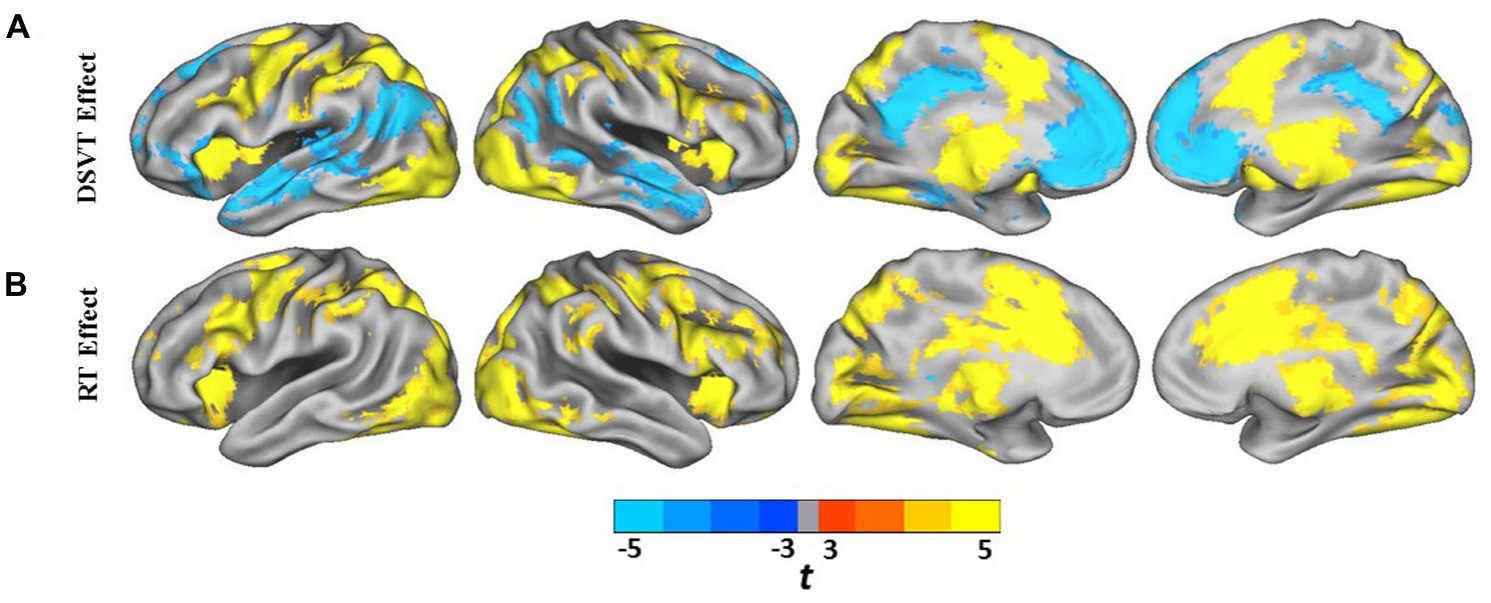
**Lateral and medial views of statistical parametric maps of DSVT effects and RT effects. (A)** Color scaled *t*-values from voxel-wise one sample *t-*tests comparing mean percent DSVT-related BOLD percent signal change to zero. **(B)** Color scaled *t*-values from voxel-wise one sample *t*-tests comparing mean RT-related signal change to zero. All voxel-wise *p*s ≤ 0.005.

**FIGURE 4:**
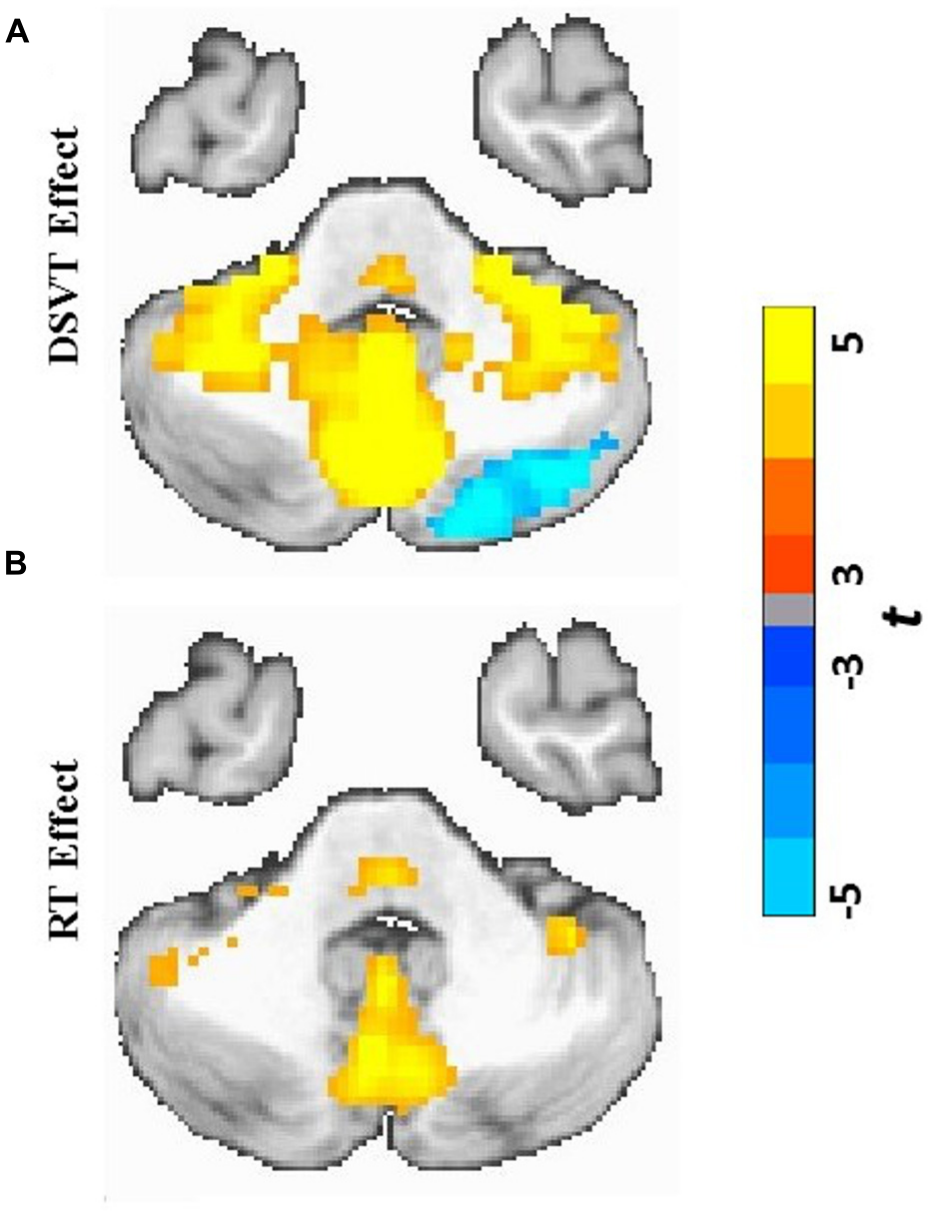
**Cerebellar and subcortical views of statistical parametric maps of DSVT effects and RT effects. (A)** Color scaled *t*-values from voxel-wise one sample *t-*tests comparing mean percent DSVT-related BOLD percent signal change to zero. **(B)** Color scaled *t*-values from voxel-wise one sample *t*-tests comparing mean RT-related signal change to zero. All voxel-wise *p*s ≤ 0.005.

Group analyses were performed on the subject-level DSVT-effect and subject-level RT-effect parameter estimates (**Figures 3A,B and 4A,B**). **Figures 3A and 4A** illustrate the performance-independent DSVT-effect BOLD percent signal changes. **Figures 3B and 4B** illustrates the performance-dependent trial-level RT-effect correlations with trial-level BOLD percent signal change, or intra-subject variability in processing speed.

#### Performance-independent DSVT effects

This analysis revealed regions of BOLD percent signal change activations and deactivations independent of subjects’ task performance.

Patterns of activation and deactivation were found in a wide range of brain regions (**Figures 3A and 4A**; see **Table 2** for focal anatomical areas). Voxel-wise one-sample *t*-tests, comparing the subject-level amplitude parameter estimates to zero, were calculated to identify significant activations and deactivations (*t*_min_[29] = -11.37 and* t*_max_[29] = 18.19; with all voxel-wise *p*s ≤ 0.005).

**Table 2:** Focal anatomical areas of significant BOLD percent signal change for the performance-independent DSVT effects (all voxel-wise *p*s ≤ 0.005).

Anatomical structure	Brodmann area	Talairach coord of peak voxel (RAI order)			Number voxels	Maximum *t* value at peak voxel	Average % signal-change (SD)
		x	y	z
Right Inferior Occiptial	18	–29	87	–2	55695	18.19	0.54 (0.16)
Left Anterior Cingulate	32	3	–19	–8	8047	–9.57	–0.15 (0.09)
Left Angular	19	43	77	30	5420	–9.97	–0.26 (0.14)
Right Middle Temporal	39	–49	69	28	3440	–9.83	–0.20 (0.11)
Left Cingulate	31	1	41	32	2306	–11.37	–0.14 (0.07)
Right Pyramis	–	–19	79	–34	391	–6.37	–0.08 (0.07)
Right Cuneus	18	–13	85	18	166	–5.30	–0.14 (0.15)

Patterns of significant activations (illustrated in red to yellow) occurred within visual regions including bilateral activation occurred in primary visual cortex (BA 17), both foveal and parafoveal regions, and extending across lateral occipital and visual association areas (BAs 18, 19, 20, and 37). Within parietal areas, bilateral activation occurred in superior parietal (BA 7) and inferior parietal (BA 40) regions. Within frontal areas, activation extended from dorsal PFC (BAs 9 and 46) to ventral PFC (BAs 44 and 47), and to insula cortex (BAs 30, 31, and 32). Additionally, bilateral activation occurred within medial PFC regions (BA 6) and cingulate cortex (BA 24). Motor activation patterns consistent with the manual responses also were detected within motor and supplementary motor regions and within the cerebellum.

Patterns of significant bilateral deactivation (illustrated in blue to cyan) also occurred within temporal areas, deactivation extended from inferior parietal regions (BA 39) along superior and middle temporal regions (BAs 21, 22) to the temporal pole (BA 38). Deactivation also occurred within areas of the default network, in anterior and posterior cingulate cortex (Gusnard and Raichle, 2001).

#### Performance-dependent trial-level RT effects

This analysis revealed regions of BOLD percent signal change variability dependent on trial-level task performance (i.e., intra-subject variability). That is, trial-level RT-BOLD correlations across subjects.

Patterns of positive and negative correlations between RT and BOLD percent signal change were found in more localized regions compared to the DSVT effects (**Figures 3B and 4B**; see **Table 3** for focal anatomical areas). Voxel-wise one-sample *t*-tests, comparing the slopes (i.e., standardized trial-level RT-BOLD Bs) relating the RT model values to BOLD percent signal change, were calculated to identify significant group-level trial-level RT-BOLD percent signal change correlations (*t*_min_[29] = -7.90 and* t*_max_[29] = 10.55; with all voxel-wise *p*s ≤ 0.005).

**Table 3:** Focal anatomical areas of significant RT-BOLD correlations for the performance-dependent trial-level RT effects (all voxel-wise *p*s ≤ 0.005).

Anatomical structure	Brodmann area	Talairach coord. of peak voxel (RAI order)			Number voxels	Maximum *t* value at peak voxel	Average slope for subj.-level RT-BOLD correlations (SD)
		x	y	z
Right cingulate	32	–7	–15	34	47717	10.55	0.75 (0.39)
Right caudate	–	–25	41	12	762	–7.90	–0.38 (0.27)
Left caudate	–	23	43	12	541	–7.60	–0.39 (0.28)
Left middle frontal	9	27	–27	26	343	4.00	0.34 (0.46)

Patterns of significant positive correlations, wherein slower RTs were associated with greater BOLD percent signal change (illustrated by red to yellow), occurred within visual areas. Trial-level RTs were associated with BOLD percent signal change within lateral occipital and visual association areas (BAs 18, 19, 20, and 37) as well as within primary visual areas (BA 17).

Positive correlations also occurred bilaterally within parietal and frontal regions. Slower RT also was associated with greater BOLD percent signal change within superior parietal (BA 7), particularly located in the intraparietal sulcus and the occipito-parietal junction, and inferior parietal cortex (BA 40). Additionally, slower RT was associated with greater BOLD percent signal change within both dorsal (BA 8), ventral (BA 46), and medial (BA 6, extending into BA 24) PFC. Faster processing speed was associated with less activation within these regions.

Patterns of significant negative correlations wherein slower RTs were associated with less BOLD percent signal change (illustrated by blue to cyan) occurred within bilateral caudate.

In regions in which patterns of significant bilateral performance-independent DSVT deactivation were observed [e.g., temporal areas, inferior parietal regions (BA 39), superior and middle temporal regions (BAs 21, 22), temporal pole (BA 38) and areas of the default network (Gusnard and Raichle, 2001)], we did not observe performance-dependent RT effects in which RT was associated with BOLD activity.

## DISCUSSION

The present study explored the brain bases of intra-subject variability in cognitive efficiency using an fMRI-adapted DSVT processing speed task. Correlations between trial-level RTs and corresponding fMRI BOLD percent signal change on the DSVT were assessed. Positive correlations, where slower RTs were associated with greater BOLD percent signal change, were observed across a more circumscribed set of regions compared to task-related BOLD effects.

Performance-independent DSVT effects (i.e., DSVT BOLD effects) were observed in primary visual cortex and secondary and associative visual areas. Additional task-related activation was observed in dorsal, ventral, and medial PFC, insula, and cingulate cortex. Deactivation was observed in temporal and inferior parietal areas as well as regions associated with the default network (Gusnard and Raichle, 2001). Task-related activation and deactivation patterns were consistent with previous findings (e.g., Rypma et al., 2006) illustrating a wide set of regions associated with processing speed.

Whereas performance-independent DSVT effects were observed in a large set of brain regions, a smaller subset of regions were involved in mediating performance-dependent DSVT effects, or intra-subject processing speed variability. Performance-dependent effects (i.e., RT-BOLD correlations) were observed in primary and secondary visual and association cortex, but not areas of the default network as observed in the performance-independent effects. These results suggest that trial-to-trial variability in processing speed emanates from a more circumscribed set of regions than task-general effects.

The absence of an association between RT and BOLD percent signal change in the default network suggests that those networks that support performance are independent of those associated with rest such as the default mode network. Other work showing associations between task-negative BOLD and performance has been taken to represent reallocation of processing resources to regions involved in performing the task (Burzynska et al., 2013). The present results are not consistent with this hypothesis. Clearly more work is needed to understand the relationships between task-related activity, rest-related activity, and performance.

In the present study, slower RTs were associated with greater PFC BOLD percent signal change. The trial-level RT-BOLD correlations are consistent with previous inter-subject observations in which slower mean RTs were associated with greater BOLD percent signal change (Motes et al., 2011) and associated with greater connectivity from PFC to other regions (e.g., Rypma et al., 2006) suggesting a PFC-related executive guidance account of inter-subject and intra-subjectdifferences in processing speed. The present intra-subject level analysis also indicated that slower RTs were associated with greater inferior and superior parietal BOLD percent signal change. Prior inter-subject variability results showed greater BOLD activity within inferior parietal regions associated with faster mean RT (Rypma et al., 2006) suggesting a functional dissociation between processing speed variability (i.e., RT variability) and mean processing speed (i.e., mean RT), and thus also between trial-level cognitive efficiency and subject-level cognitive efficiency.

Several lines of evidence support the hypothesis that RT-BOLD correlations reflect variability in processing speed at the inter-subject level. A strength of the present study is the use of a direct measure of processing speed to observe intra-subject cognitive efficiency effects. DSVT performance correlates with performance on other independent measures of processing speed (i.e., Digit Symbol Coding; Wechsler, 1985; see also Rypma et al., 2006; **Table 1**), providing evidence that the DSVT is a valid measure of processing speed. Processing-speed measures also account for both juvenile and adult developmental performance differences (see Kail, 1991; Salthouse, 1996) across a wide range of cognitive tasks. Furthermore, variability in processing speed also accounts for variability in performance on Raven’s Progressive Matrices and other measures of *g* (Barrett et al., 1990; Jensen, 1992a) associated with neural activity integration of parieto-frontal regions [see parieto-frontal integration theory (P-FIT); Jung and Haier, 2007]. The P-FIT network explains the brain regions, connected by white-matter pathways, underpinning individual differences in reasoning competence and intelligence (Jung and Haier, 2007).

The present results, using a basic measure of processing speed, suggests that RT-BOLD correlations distributed across a broad set of regions might reflect the effects of task-general, processing speed variability. Longer DSVT RTs were generally associated with greater BOLD percent signal change across frontal, parietal, and caudate regions (in addition to visual regions and insula) overlapping with areas showing task-general correspondence in RT-BOLD correlations. In particular, trial-to-trial variability in processing speed might underlie the task-general correspondence in RT-BOLD correlations reported in other studies (Rypma et al., 2006).

Our data support the hypothesis of a set of regions, including the PFC and parietal cortex, that mediates trial-to-trial intra-subject performance variability. Other psychological mechanisms might additionally contribute to intra-subject performance variability. Some theorists have posited that individual differences in cognitive strategies can contribute to individual differences in efficient cognitive performance. On the Sternberg working memory task for instance, it has been suggested that some individuals place more emphasis on encoding whereas others place more emphasis on maintenance or retrieval processes to successfully execute a working memory trial (Wagner and Sternberg, 1985; Braver et al., 2009). Differential BOLD signal amplitude in one task period or another is thought to reflect such strategy differences. Previous neuroimaging research suggests evidence for task-period-specific individual differences in neural activity (Rypma et al., 2002; Braver et al., 2009). Individual variability in the BOLD amplitude difference between less-demanding and more-demanding task conditions has also been posited to reflect strategy differences (e.g., Rypma and D’Esposito, 1999). For instance, during the delay period of trials within a working memory task, faster performers showed greater PFC BOLD signal amplitude than slower performers on a Sternberg item-recognition task (Rypma et al., 2007). Thus it might be that faster performers might strategically utilize the time between encoding and retrieval to perform additional processing aimed at optimizing performance. Faster performers might also utilize the time between trials to carry out similar performance-optimizing strategies. In fact, pre-trial RT-BOLD correlations are observed across a wide range of cognitive tasks that have indirectly measured working memory, episodic memory, decision-making, and affective rating (Weissman et al., 2006). Decreased PFC BOLD percent signal change, prior to a trial, were associated with longer RTs (Weissman et al., 2006) and increased PFC BOLD percent signal change, during a trial, were associated with faster RTs (Yarkoni et al., 2009), suggesting strategic attentional preparation in advance of the trial and goal-directed maintenance of attention during the trial (see Burgess et al., 2011). Rest-related activity might also facilitate strategic inter-trial processing which may aid in development of more efficient, less executive-demanding “automatic,” task-performance. More research is certainly needed to understand how physiological networks and psychological processes interact in the service of optimizing task performance.

The results reported in the present study suggest that an organized set of brain regions are associated with intra-subject processing-speed variability. We observed a broad-spread DSVT-related regions revealed by performance-independent activation (and deactivation). We also observed a broad, but more restricted, DSVT performance-dependent brain regions in which greater activation was associated with slower RT, and these cognitive efficiency patterns occurred in regions showing positive DSVT-related BOLD signal changes. These results support the hypothesis that a subset of task-related processing regions are associated with intra-subject cognitive efficiency variability and might contribute to the individual variability observed in processing speed measures contributing to individual variability in general intelligence.

## Conflict of Interest Statement

The authors declare that the research was conducted in the absence of any commercial or financial relationships that could be construed as a potential conflict of interest.
